# MAPK13 stabilization *via* m^6^A mRNA modification limits anticancer efficacy of rapamycin

**DOI:** 10.1016/j.jbc.2023.105175

**Published:** 2023-08-19

**Authors:** Joohwan Kim, Yujin Chun, Cuauhtemoc B. Ramirez, Lauren A. Hoffner, Sunhee Jung, Ki-Hong Jang, Varvara I. Rubtsova, Cholsoon Jang, Gina Lee

**Affiliations:** 1 Department of Microbiology and Molecular Genetics, Chao Family Comprehensive Cancer Center, School of Medicine, University of California Irvine, Irvine, California, USA; 2 Department of Biological Chemistry, Chao Family Comprehensive Cancer Center, School of Medicine, University of California Irvine, Irvine, California, USA; 3 School of Biological Sciences, University of California Irvine, Irvine, California, USA

**Keywords:** m^6^A, RNA modification, RNA stability, MAPK13, p38, mTORC1, rapamycin

## Abstract

*N*^6^-adenosine methylation (m^6^A) is the most abundant mRNA modification that controls gene expression through diverse mechanisms. Accordingly, m^6^A-dependent regulation of oncogenes and tumor suppressors contributes to tumor development. However, the role of m^6^A-mediated gene regulation upon drug treatment or resistance is poorly understood. Here, we report that m^6^A modification of mitogen-activated protein kinase 13 (*MAPK13*) mRNA determines the sensitivity of cancer cells to the mechanistic target of rapamycin complex 1 (mTORC1)-targeting agent rapamycin. mTORC1 induces m^6^A modification of *MAPK13* mRNA at its 3′ untranslated region through the methyltransferase-like 3 (METTL3)–METTL14–Wilms' tumor 1–associating protein(WTAP) methyltransferase complex, facilitating its mRNA degradation *via* an m^6^A reader protein YTH domain family protein 2. Rapamycin blunts this process and stabilizes *MAPK13*. On the other hand, genetic or pharmacological inhibition of *MAPK13* enhances rapamycin’s anticancer effects, which suggests that *MAPK13* confers a progrowth signal upon rapamycin treatment, thereby limiting rapamycin efficacy. Together, our data indicate that rapamycin-mediated *MAPK13* mRNA stabilization underlies drug resistance, and it should be considered as a promising therapeutic target to sensitize cancer cells to rapamycin.

Transcription and translation are central mechanisms to control gene expression. In addition to these canonical processes, cells modify genetic materials with various chemical moieties as an additional layer of gene regulation. While epigenetic modifications of DNA and histones are well established, chemical modifications of RNA (*i.e.*, epitranscriptomic regulation) have been recently shown to play crucial roles in gene regulation (1, 2). Of the mRNA modifications, m^6^A is the most abundant (3). m^6^A is deposited on mRNA by a methyltransferase complex, which is composed of three core proteins: methyltransferase-like 3 (METTL3), METTL14, and Wilms' tumor 1–associating protein (WTAP) (4, 5). m^6^A is mostly enriched on the last exon of mRNA near the stop codon and 3′UTR as revealed by transcriptome-wide sequencing (6, 7). These m^6^A-modified mRNAs then recruit m^6^A-binding “reader” proteins that determine the diverse fates of these mRNAs. For example, the YTHDF (YTH domain family) of m^6^A reader proteins decrease stability or promote the translation efficiency of m^6^A-containing mRNAs (8, 9).

m^6^A-dependent gene regulation is involved in diverse biological processes, such as embryo development, stem cell differentiation, sex determination, and circadian rhythm; dysregulation of this process can cause various diseases including cancers (10, 11, 12). Interestingly, both increased and decreased m^6^A levels can lead to cancer development, depending on the downstream target genes. METTL3 overexpression in leukemia cells induces expression of oncogenes such as *cMyc* and *Bcl2* (13). On the other hand, METTL3 downregulation in endometrial cancer induces Akt prosurvival signaling by decreasing the expression of Akt inhibitor, PHLPP2 (PH domain and leucine-rich repeat protein phosphatase 2) (14). Therefore, a comprehensive examination of m^6^A target genes is necessary to better understand the impact of m^6^A modification in different biological and pathological contexts.

As a master regulator of cell growth, mechanistic target of rapamycin complex 1 (mTORC1) is overactivated in most human cancers (15, 16, 17, 18, 19, 20). The mTORC1 inhibitor rapamycin was considered as a promising therapeutic agent, but it faced several clinical challenges such as drug resistance or regrowth of tumors after treatment (21, 22, 23). It has been suggested that mTORC1-dependent post-translational modification of proteins (*e.g.*, protein phosphorylation) underlie the observed rapamycin resistance mechanisms. However, whether post-transcriptional RNA modifications confer rapamycin resistance is unknown.

Recent work from our and other laboratories revealed that activation of m^6^A mRNA modification by mTORC1 contributes to tumor progression. mTORC1 induces expression of METTL3, METTL14, and WTAP, which methylates and destabilizes the growth-suppressing genes such as cMyc suppressor and autophagy genes (24, 25, 26, 27). From our transcriptome-wide m^6^A sequencing, we identified additional target genes that are potentially regulated by mTORC1-dependent m^6^A modification (24). In this study, we report that a mitogen-activated protein kinase (MAPK)/p38 isoform, MAPK13/p38δ, is a downstream target of the mTORC1–m^6^A RNA modification pathway, which likely contributes to the limited tumor-suppressive effects of rapamycin.

## Results

### Identification of genes regulated by mTORC1 and m^6^A writer complex

**Figure 1:**
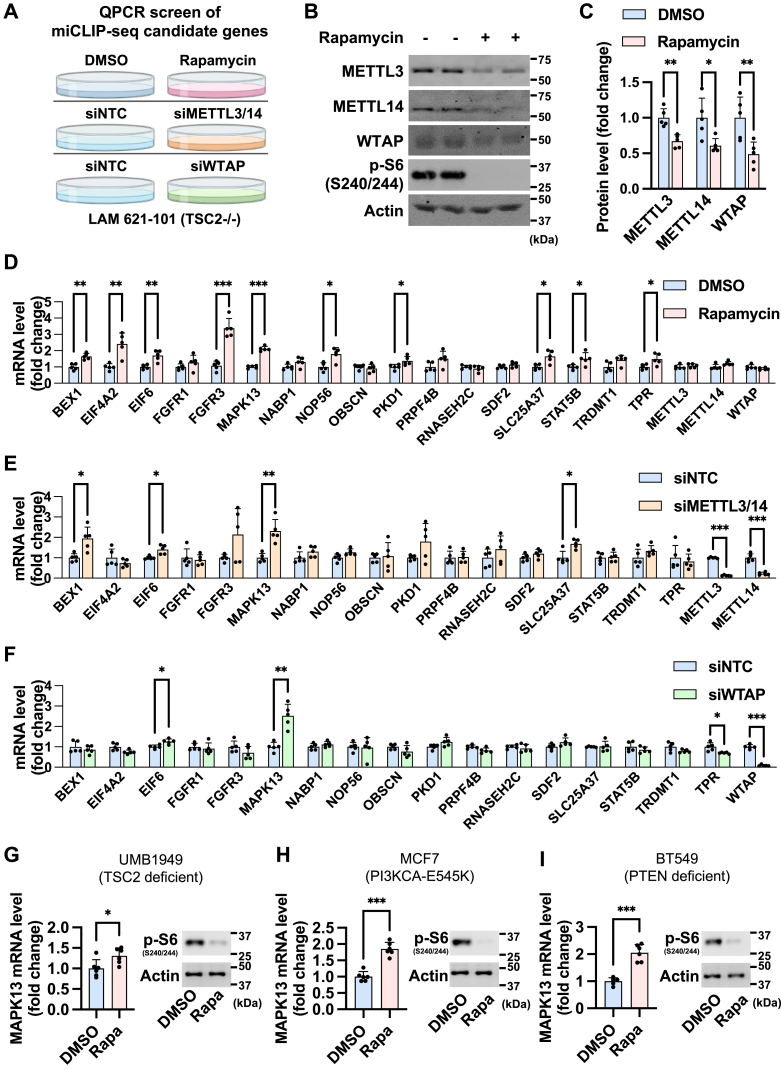
**Identification of *MAPK13* as the downstream target of rapamycin and m**^**6**^**A writer complex.***A*, schematic of the qPCR screen in LAM 621-101 (*TSC2*^*−/−*^) cells to identify target genes regulated by rapamycin and m^6^A writer complex. The screen sets include three conditions treated with DMSO (control) *versus* rapamycin for 48 h, transfected with siNTC (control) *versus* siMETTL3/14, and transfected with siNTC *versus* siWTAP. Candidate genes were selected from our previous miCLIP-Seq in HEK293E cells treated with mTOR inhibitor, torin1 (24). *B* and *C*, immunoblot analysis of LAM 621-101 cells treated with DMSO or rapamycin. *C*, a quantification graph of immunoblot bands. N = 5. *D*–*F*, qPCR analysis of 17 candidate genes in LAM 621-101 cells treated with DMSO *versus* rapamycin (*D*), transfected with siNTC *versus* siMETTL3/14 (*E*), or transfected with siNTC *versus* siWTAP (*F*). N = 5. *G*–*I*, qPCR and immunoblot analyses of UMB1949 (*G*), MCF7 (*H*), and BT549 (*I*) cells treated with DMSO or rapamycin. ∗*p* < 0.05, ∗∗*p* < 0.01, ∗∗∗*p* < 0.001. Error bars show SD. Numbers on the immunoblot indicate the positions of molecular weight markers. See also Fig. S1. DMSO, dimethyl sulfoxide; HEK293E, human embryonic kidney 293E cell line; LAM, lymphangioleiomyomatosis; m^6^A, *N*^6^-adenosine methylation; *MAPK13*, mitogen-activated protein kinase 13; METTL, methyltransferase-like protein; miCLIP, m6A individual-nucleotide-resolution crosslinking and immunoprecipitation; mTOR, mechanistic target of rapamycin; qPCR, quantitative PCR; TSC2, tuberous sclerosis complex 2; WTAP, Wilms' tumor 1–associating protein.

We previously performed m^6^A individual-nucleotide-resolution crosslinking and immunoprecipitation (miCLIP)-Seq in human embryonic kidney 293E (HEK293E) cells, identifying the 17 genes whose m^6^A level is decreased, whereas total mRNA expression is increased by the mTOR catalytic inhibitor, torin1 (24). Since torin1 suppresses both mTORC1 and mTORC2, we then used rapamycin to selectively block mTORC1 and performed quantitative PCR (qPCR) analysis as a secondary screen of candidate genes identified from miCLIP-Seq (Fig. 1*A*). In parallel, we depleted m^6^A writer complex proteins, METTL3/14 or WTAP, to validate the genes that are regulated by m^6^A modification. For these screens, we used lymphangioleiomyomatosis (LAM) 621-101 cell line, a kidney angiomyolipoma cell line isolated from an LAM patient. LAM 621-101 cells have an overactive mTORC1 activity because of a loss of function in the tumor suppressor protein called tuberous sclerosis complex 2 (TSC2) (28, 29). Consistent with our previous findings, inhibition of mTORC1 activity by rapamycin reduced the protein levels of m^6^A writer proteins METTL3, METTL14, and WTAP (Fig. 1, *B* and *C*) (24, 25). We found ten genes (*BEX1* [brain expressed X-linked 1], *EIF4A2* [eukaryotic translation initiation factor 4A2], *EIF6* [eukaryotic translation initiation factor 6], *FGFR3* [fibroblast growth factor receptor], *MAPK13*, *NOP56* [NOP56 ribonucleoprotein], *PKD1* [polycystic kidney disease 1], *SLC25A37* [solute carrier family 25 member 37], *STAT5B* [signal transducer and activator of transcription 5B], and *TPR* [translocated promoter region]) whose mRNA levels were elevated by rapamycin (Fig. 1*D*). *METTL3/14* knockdown increased mRNA levels of *BEX1*, *EIF6*, *MAPK13*, and *SLC25A37* (Fig. 1*E*), and *WTAP* knockdown increased mRNA levels of *EIF6* and *MAPK13* (Fig. 1*F*). Analysis of published Gene Expression Omnibus (GEO) dataset (GSE193402) revealed that rapamycin induces mRNA levels of *MAPK13*, *OBSCN* [obscurin], *SLC25A37*, *and STAT5B* in another TSC2-deficient renal angiomyolipoma cell line, UMB1949 (30, 31) (Fig. S1). qPCR analysis further validated *MAPK13* induction upon rapamycin treatment in several mTORC1-overactive cells including UMB1949, MCF7 (PI3K-mutated breast cancer) (32), and BT549 (PTEN-deficient breast cancer) (33) (Fig. 1, *G*–*I*). Thus, we decided to further study *MAPK13* based on its dramatic and consistent induction in all conditions across diverse cancer cells.

### m^6^A writer complex regulates MAPK13/p38δ expression among p38 isoforms

**Figure 2:**
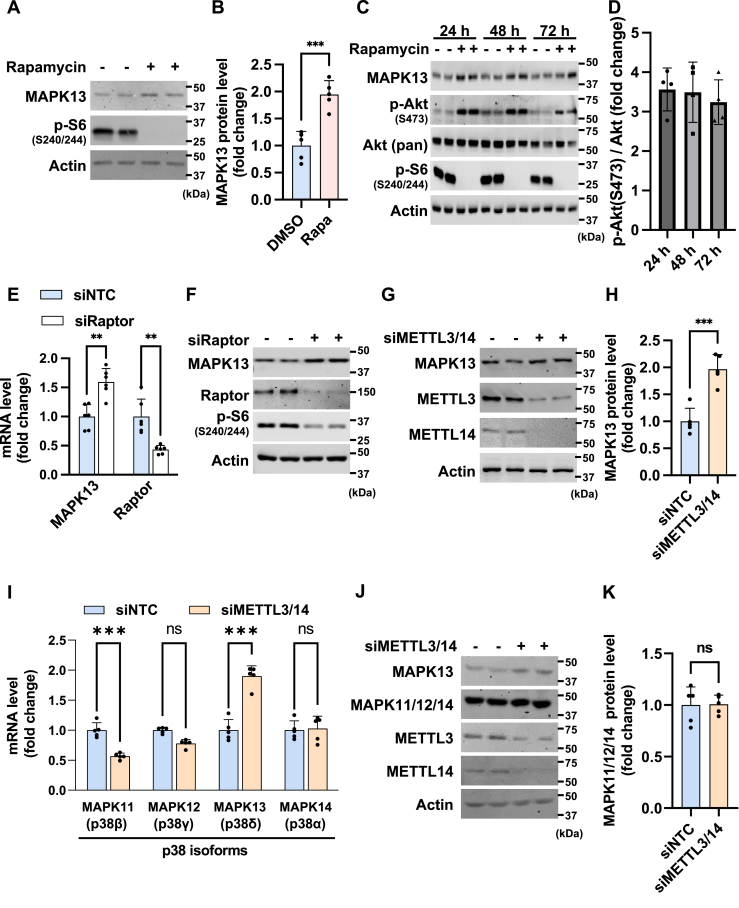
**mTORC1 and m**^**6**^**A regulate MAPK13/p38δ expression among p38 MAPK isoforms.***A* and *B*, immunoblot analysis of LAM 621-101 cells treated with DMSO or rapamycin. *B*, the quantification graph of immunoblot bands. N = 5. *C* and *D*, immunoblot analysis of LAM 621-101 cells treated with rapamycin in time course. *D*, a quantification graph of immunoblot bands. N = 5. *E* and *F*, qPCR analysis (*E*) and immunoblot analysis (*F*) of LAM 621-101 cells transfected with siNTC or siRaptor. N = 5. *G* and *H*, immunoblot analysis of LAM 621-101 cells transfected with siNTC or siMETTL3/14. *D*, a quantification graph of immunoblot bands. N = 5. *I*, qPCR analysis of p38 MAPK family genes. LAM 621-101 cells were transfected with siNTC or siMETTL3/14. N = 5. *J* and *K*, immunoblot analysis of LAM 621-101 cells transfected with siNTC or siMETTL3/14. *G*, a quantification graph of immunoblot bands. N = 5. ∗∗∗*p* < 0.001, ns = not significant. Error bars show SD. Numbers on the immunoblot indicate the positions of molecular weight markers. DMSO, dimethyl sulfoxide; LAM, lymphangioleiomyomatosis; m^6^A, *N*^6^-adenosine methylation; MAPK13, mitogen-activated protein kinase 13; mTORC1, mechanistic target of rapamycin complex 1; qPCR, quantitative PCR.

Next, we assessed protein levels of MAPK13 to examine whether the changes in *MAPK13* mRNA levels are reflected in MAPK13 protein expression. Upon rapamycin treatment, the protein levels of MAPK13 increased by twofold (Fig. 2, *A* and *B*). Since rapamycin has been shown to suppress both mTORC1 and mTORC2 in some conditions (34, 35, 36), we looked at mTORC2 activity using Akt-S473 phosphorylation as a readout. In contrast, the near-complete suppression of mTORC1 activity (measured by pS6-S240/S244) by rapamycin, mTORC2 activity (measured by pAkt-S473) was not inhibited by rapamycin in LAM 621-101 cells (Fig. 2, *C* and *D*). Rapamycin rather induced Akt phosphorylation (Fig. 2, *C* and *D*), indicating the release of negative feedback suppression of mTORC2 by mTORC1 upon rapamycin treatment (23, 37). Knockdown of *Raptor*, a key component of mTORC1 complex, increased MAPK13 mRNA and protein levels (Fig. 2, *E* and *F*), demonstrating mTORC1-dependent regulation of MAPK13 expression. Finally, double knockdown of *METTL3/14* also led to twofold increase in MAPK13 protein expression (Fig. 2, *G* and *H*). Overall, the extent of MAPK13 protein induction (Fig. 2, *A*–*H*) correlated well with the increase in its mRNA levels (Fig. 1, *D*–*F*).

MAPK13 is a member of the p38 MAPK protein family composed of p38α (MAPK14), p38β (MAPK11), p38γ (MAPK12), and p38δ (MAPK13). These proteins control diverse cellular signaling processes, including proliferation, differentiation, inflammation, and cell death responses (38, 39, 40, 41, 42). Interestingly, in contrast to *MAPK13*, knockdown of *METTL3/14* did not induce mRNA expression of *MAPK11*, *MAPK12*, or *MAPK14* (Fig. 2*I*). In the case of *MAPK11*, its mRNA level was decreased (Fig. 2*I*). To examine protein level changes of these MAPK isoforms, we used an antibody that detects amino acid sequences across three p38 MAPK isoforms, MAPK11, MAPK12, and MAPK14. This antibody does not detect MAPK13 (43). Interestingly, the protein expression of these p38 isoforms (MAPK11, MAPK12, and MAPK14) did not change regardless of *METTL3/14* knockdown (Fig. 2, *J* and *K*). Thus, MAPK13 is a unique p38 isoform suppressed by mTORC1-dependent m^6^A modification.

### mTORC1–m^6^A–YTHDF2 destabilizes *MAPK13* mRNA

**Figure 3:**
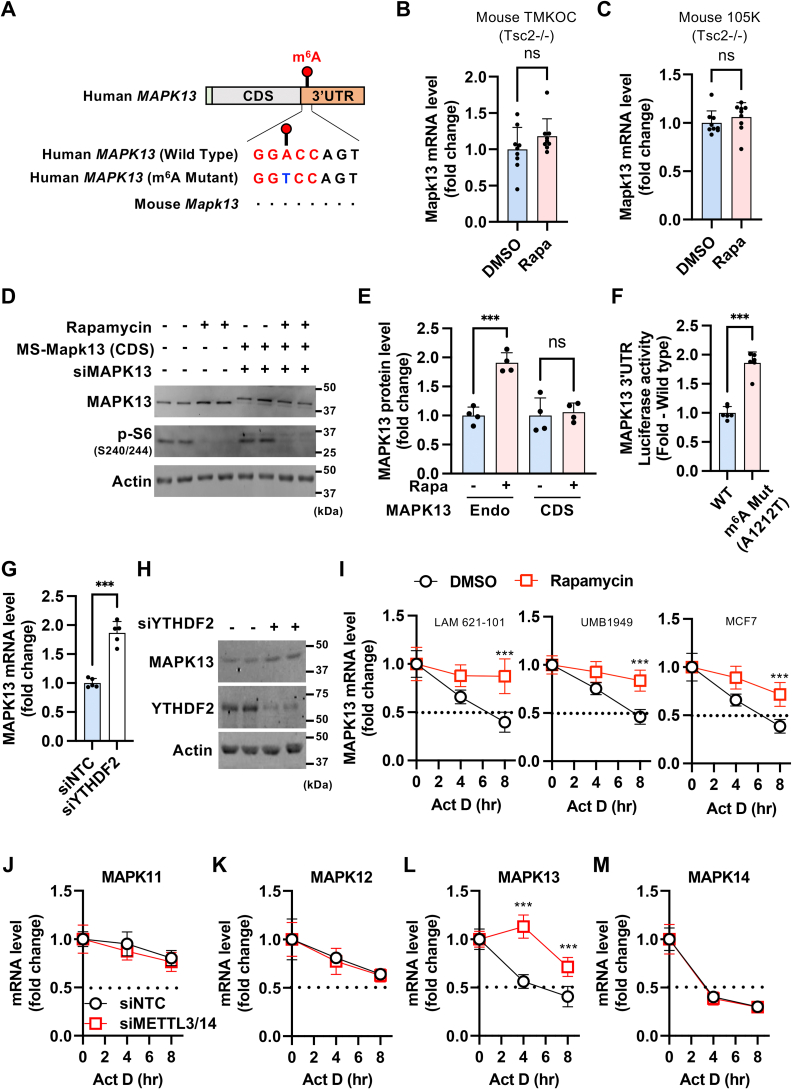
***MAPK13*mRNA stability is regulated by mTORC1–m**^**6**^**A–YTHDF2 axis.***A*, schematic of human *MAPK13* mRNA containing m^6^A modification site on the 3′UTR. Sequence alignment analysis revealed that the m^6^A modification site is not conversed in mouse *Mapk13*. Detailed sequence conservation analysis is shown in Fig. S2. In the m^6^A mutant construct, A1212 was mutated to T in the 3′UTR of human *MAPK13*. *B* and *C*, qPCR analysis of *Mapk13* mRNA levels in mouse TMKOC (*B*) and 105K (*C*) cells treated with DMSO or rapamycin. N = 9. *D* and *E*, immunoblot analysis of LAM 621-101 cells treated with DMSO or rapamycin. Endogenous *MAPK13* (Endo) was knocked down with siRNA, and siRNA-resistant mouse *Mapk13* (CDS) was ectopically expressed. *E*, quantification of MAPK13 protein expression normalized to DMSO-treated group in each condition. N = 4. *F*, luciferase activity of *MAPK13* 3′UTR renilla luciferase reporters containing WT or mutant (m^6^A Mut, A1212T) m^6^A sites. The renilla luciferase activity was normalized to control cypridina luciferase activity. N = 6. *G* and *H*, qPCR (N = 5) (*G*) and immunoblot analysis (*H*) of LAM 621-101 cells transfected with siNTC or siYTHDF2. *I*, mRNA stability analysis of *MAPK13* in LAM 621-101, UMB1949, and MCF7 cells treated with rapamycin. Cells were treated with actinomycin D for the indicated times, and qPCR was performed to measure the remaining mRNA level. N = 5. *J*–*M*, mRNA stability analysis of *p38* isoform in LAM 621-101 cells transfected with siNTC or siMETTL3/14. Cells were treated with actinomycin D for the indicated times, and qPCR was performed to measure the remaining mRNA level. N = 5. ∗*p* < 0.05, ∗∗*p* < 0.01, ∗∗∗*p* < 0.001. Error bars show SD. Numbers on the immunoblot indicate the positions of molecular weight markers. See also Fig. S2. 3′UTR, 3′ untranslated region; CDS, coding sequence; DMSO, dimethyl sulfoxide; m^6^A, *N*^6^-adenosine methylation; *MAPK13*, mitogen-activated protein kinase 13; mTORC1, mechanistic target of rapamycin complex 1; qPCR, quantitative PCR; YTHDF2, YTH domain family protein 2.

From the analysis of our previous miCLIP-Seq in human HEK293E cells (24), we found an mTORC1-dependent m^6^A modification site on the 3′UTR of *MAPK13* (Fig. 3*A*). Because some m^6^A modification sites have been shown to be conserved in between human and mouse (6, 7, 44, 45), we investigated whether *MAPK13* m^6^A modification is also conserved in mice. Interestingly, although the mouse *Mapk13* had a well-conserved coding sequence (CDS) with human *MAPK13* (92% homology), the 3′UTR (57.7% homology) and m^6^A site were not conserved in mouse *Mapk13* (Figs. 3*A* and S2). Rapamycin did not induce *Mapk13* expression in TSC2-deficient mouse kidney tumor cell lines (Fig. 3, *B* and *C*), indicating the lack of mTORC1 and m^6^A-dependent MAPK13 regulation mechanisms in mice.

To validate m^6^A-dependent regulation of *MAPK13*, we utilized the mouse *Mapk13* CDS expression construct (42) that is resistant to human *MAPK13* siRNA. This plasmid enabled the expression of *Mapk13* CDS in human cells knocked down with endogenous *MAPK13* (Fig. 3*D*). Rapamycin selectively increased expression of the m^6^A site containing endogenous *MAPK13* but not the one that lacks m^6^A modification site (*Mapk13* CDS) (Fig. 3, *D* and *E*). Furthermore, a luciferase assay revealed that abrogation of the m^6^A modification site (A1212 to T mutation) increases expression of *MAPK13* 3′UTR luciferase reporter (Fig. 3, *A* and *F*). These results indicate that m^6^A modification on *MAPK13* 3′UTR decreases its expression, and rapamycin reverses this regulatory process by suppressing the mTORC1-dependent m^6^A modification.

Once modified with m^6^A, mRNAs recruit m^6^A reader proteins that determine the fate of target transcripts such as changes in mRNA stability or translation efficiency (8, 9). Given that suppression of such m^6^A modification on *MAPK13* by *METTL3/14* or *WTAP* knockdown increases both MAPK13 mRNA and protein levels (Figs. 1 and 2), we hypothesized that *MAPK13* mRNA is degraded by YTHDF2, an m^6^A reader protein that destabilizes target transcripts (8, 9, 46, 47). Consistent with our hypothesis, knockdown of *YTHDF2* resulted in a significant increase in *MAPK13* mRNA levels (Fig. 3*G*). Consequently, MAPK13 protein levels also increased (Fig. 3*H*). Hence, YTHDF2 is the effector protein responsible for *MAPK13* mRNA degradation upon mTORC1-mediated m^6^A modification.

To further verify whether the stability of *MAPK13* mRNA is indeed regulated by mTORC1-dependent m^6^A modification, we assessed mRNA half-life. To this end, we treated several cancer cell lines with actinomycin D to block *de novo* mRNA synthesis and measured the remaining transcript levels at different time points (48). In the vehicle-treated control condition, *MAPK13* mRNA was degraded in a time-dependent manner with a half-life of 6 to 8 h (Fig. 3*I*). However, upon rapamycin treatment, the stability of *MAPK13* mRNA was dramatically increased, with 75 to 90% of transcripts remaining even after 8 h (Fig. 3*I*). Similarly, *METTL3/14* double knockdown also markedly increased the half-life of *MAPK13* mRNA but not that of the other three p38 isoforms (Fig. 3, *J*–*M*). Collectively, these results demonstrate that rapamycin increases mRNA stability of *MAPK13 via* the m^6^A–YTHDF2 axis.

### MAPK13 inhibition enhances rapamycin’s anticancer effect

**Figure 4:**
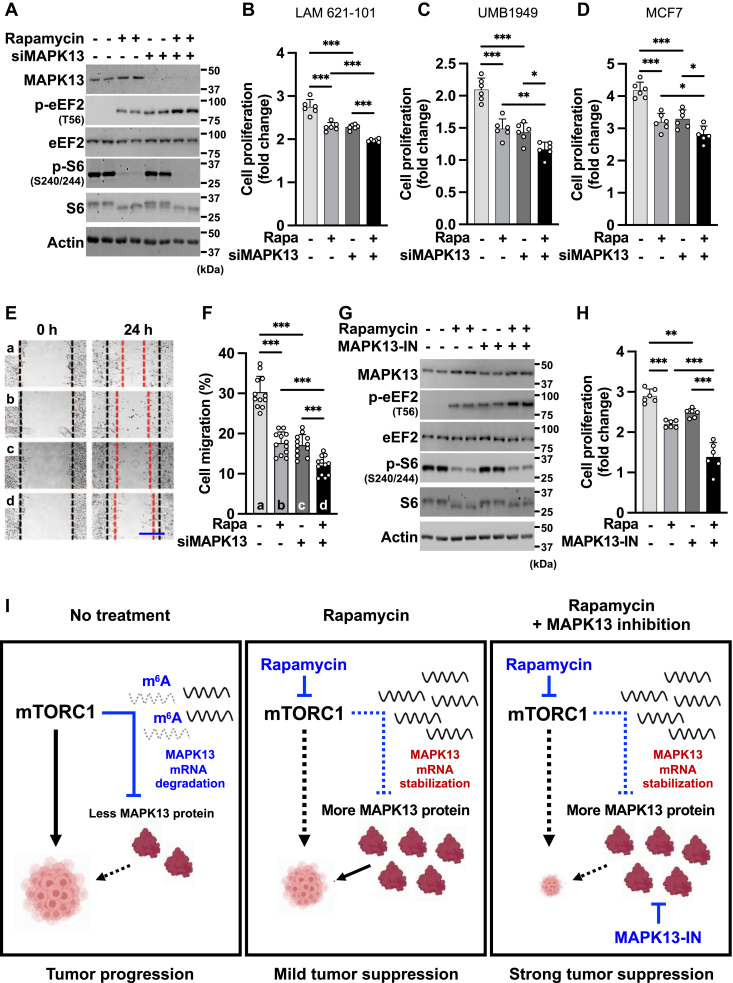
**MAPK13 inhibition enhances rapamycin’s suppressive effect on cell growth and migration.***A*, immunoblot analysis of LAM 621-101 cells transfected with siNTC or siMAPK13 in combination with DMSO or rapamycin treatment. *B*–*D*, cell proliferation assay of LAM 621-101 (*B*), UMB1949 (*C*), and MCF7 (*D*) cells transfected with siNTC or siMAPK13 in combination with DMSO or rapamycin. The graph shows the fold increase in cell numbers 3 days after drug treatment. N = 6. *E* and *F*, wound healing assay of LAM 621-101 cells transfected with siNTC or siMAPK13 in combination with DMSO or rapamycin. After scratching the cell layer to form a wound, images were captured at 0 and 24 h to assess cell migration. *Black dotted lines* indicate the initial wound area at 0 h; *red dotted lines* mark the migrating front of cells at 24 h (*E*). Cell migration efficiency was calculated by measuring the wound area at each time point by ImageJ software (*F*). Scale bar represents 500 μm. N = 12. *G* and *H*, immunoblot (*G*) and cell proliferation (*H*) analysis of LAM 621-101 cells treated with MAPK13-IN (MAPK13-IN-1, MAPK13 inhibitor) with or without rapamycin. The graph in (*H*) shows relative fold increase in cell numbers 3 days after drug treatment. N = 6. *I*, a schematic diagram describing the regulation of *MAPK13* expression by mTORC1-dependent m^6^A methylation (*left*, without rapamycin; *middle*, with rapamycin) and the synergistic effect of MAPK13 inhibition in tumor suppression in combination with rapamycin treatment (*right*). ∗*p* < 0.05, ∗∗*p* < 0.01, ∗∗∗*p* < 0.001. Error bars show SD. Numbers on the immunoblot indicate the positions of molecular weight markers. See also Fig. S3; DMSO, dimethyl sulfoxide; MAPK13, mitogen-activated protein kinase 13; mTORC1, mechanistic target of rapamycin complex 1.

Among the various MAPK family proteins, MAPK13 has been shown to contribute to tumor progression and inflammatory responses (38, 39, 40, 41, 42, 49). One such MAPK13 downstream is the eukaryotic elongation factor-2 kinase (eEF2K)–eEF2 pathway. eEF2 is a translation elongation factor that promotes translocation of peptidyl-tRNA in ribosomes, whereas eEF2K is a negative regulator of protein translation by suppressing eEF2 activity through eEF2–T56 phosphorylation (49, 50). MAPK13 phosphorylates eEF2K at Ser359 and inhibits its activity, which results in decreased eEF2 phosphorylation and enhanced protein synthesis (51, 52). Consistent with the previous reports, *MAPK13* knockdown increased eEF2–T56 phosphorylation (Figs. 4*A* and S3*A*), reflecting the enhanced eEF2K activity upon MAPK13 inhibition. It is noteworthy that eEF2K can also be suppressed by mTORC1 and its downstream effector S6K (53). Consequently, rapamycin treatment led to eEF2 phosphorylation. However, when we knocked down *MAPK13* in rapamycin-treated cells, eEF2–T56 phosphorylation was further enhanced (Fig. 4*A*), indicating that the increased expression of MAPK13 was limiting the extent of eEF2K-dependent eEF2 phosphorylation in rapamycin-treated cells.

Next, we examined the impact of rapamycin–MAPK13 signaling in cell proliferation and survival. Even though the single treatment of rapamycin or *MAPK13* knockdown reduced the proliferation of mTORC1-hyperactive cancer cells including LAM 621-101, UMB1949, and MCF7, rapamycin was more effective in cell growth suppression when MAPK13 was depleted (Fig. 4, *B*–*D*). Rapamycin-mediated cell migration suppression was also further enhanced by *MAPK13* knockdown (Fig. 4, *E* and *F*). Finally, a small molecule inhibitor of MAPK13, MAPK13-IN-1 (54, 55), also showed a synergistic effect with rapamycin in suppressing cell growth (Figs. 4, *G* and *H* and S3*B*). Together, these findings indicate that MAPK13 induction by rapamycin limits the tumor-suppressive effects of rapamycin, and the combinatory treatment of rapamycin with MAPK13 inhibitor can be more effective in impairing tumor growth compared with the rapamycin monotherapy (Fig. 4*I*).

## Discussion

Because of rapamycin’s specific inhibitory activity on mTORC1, it was initially discussed as a ground-breaking anticancer therapeutic for a broad spectrum of mTORC1-overactivated human cancers. However, clinical trials revealed that rapamycin was not as efficient as expected. Some tumors even regrow into a bigger size after cessation of rapamycin treatment, and sustained rapamycin therapies generate significant toxicities in some patients (21, 22, 23). One of the mechanisms for rapamycin resistance is activation of other growth-promoting signaling pathways (56, 57). In breast cancer patients, mitogenic extracellular signal–regulated kinase –MAPK signaling was increased in cancer tissues upon rapamycin treatment (58). This unexpected observation led to the identification of negative feedback signaling pathways downstream of mTORC1; while mTORC1 promotes anabolic pathways for cell growth, it ironically inhibits several progrowth signals including PI3K, Ras, and MEK (23). Some of these progrowth signals such as PI3K and RAS are upstream activators of mTORC1; therefore, when mTORC1 is suppressed by rapamycin, these negative feedbacks are released, resulting in the continued growth of cancer cells (59). On the other hand, cotreatment of rapamycin with PI3K or MEK inhibitors is more effective for tumor suppression in cell culture and mouse models (58, 60). Here, we identified MAPK13 as a target gene regulated by mTORC1-dependent m^6^A regulation and as another key factor that potentially limits rapamycin’s tumor-suppressive effects. MAPK13 has been shown to activate mTORC1, indicating a potential negative feedback loop between MAPK13 and mTORC1 (61). Indeed, genetic knockdown or pharmacological inhibition of MAPK13 in combination with rapamycin enhanced rapamycin’s effect on cell growth and migration suppression, suggesting MAPK13 as a promising therapeutic target for augmenting rapamycin sensitivity (Fig. 4).

In the basal state of mTORC1-overactive cells, *MAPK13* mRNA undergoes destabilization because of mTORC1-dependent m^6^A modification. However, mRNA destabilization does not completely deplete *MAPK13*, in contrast to the near-complete removal of *MAPK13* mRNA by siRNA treatment (Fig. S3*A*). Subsequently, these residual *MAPK13* mRNAs produce MAPK13 proteins. Through a cycloheximide protein stability assay, we found that MAPK13 protein exhibits remarkable stability, with a half-life exceeding 24 h. This is in stark contrast to the positive control of cycloheximide assay, cMYC, which displays a half-life of less than 1 h (Fig. S3, *C* and *D*). Building upon this observation, we propose that MAPK13 proteins synthesized from the residual *MAPK13* mRNAs maintain a minimal yet significant level of MAPK13 signaling activity under basal conditions (Fig. 4*I*, *left*). This model is further supported by the fact that genetic knockdown or small-molecule inhibitor of MAPK13 diminishes cell proliferation and attenuates MAPK13 downstream signaling (Fig. 4, *A* and *G*). On the other hand, upon rapamycin treatment, the stabilized *MAPK13* mRNAs produce even more MAPK13 proteins, which facilitates MAPK13-dependent progrowth signaling (Fig. 4*I*, *middle*). Consequently, inhibition of MAPK13 activity in conjunction with rapamycin offers the most effective tumor suppression (Fig. 4*I*, *right*).

MAPK13 is one of the four p38 MAPK family proteins. Among the isoforms, MAPK14/p38α and MAPK11/p38β are expressed in most cell types, whereas the other MAPK family genes are expressed in specific tissues; MAPK12/p38γ is expressed in the skeletal muscle, whereas MAPK13/p38δ is expressed in the kidney and lung (41, 62). Intriguingly, the kidney and lung are the two dominant organs that develop tumors in TSC and LAM patients with overactive mTORC1 activity (63). Our data indicate that, among the p38 MAPK family genes, only MAPK13/p38δ was regulated by mTORC1-dependent m^6^A modification (Figs. 2 and 3). Therefore, small-molecule inhibitors that specifically target MAPK13/p38δ isoform such as MAPK13-IN-1 can be a selective therapeutic regimen with improved efficacy and lower toxicity. While p38α/MAPK14 isoform has been most extensively studied, p38δ/MAPK13 has recently emerged as a potential drug target because of its roles in stress responses, cytokine production, and tumor development (39, 40, 55, 64). Our findings therefore highlight MAPK13 as a promising target for combination therapy with rapamycin to overcome the limited tumor suppression efficacy of rapamycin.

## Experimental procedures

### Cell culture and drug treatment

TSC2-deficient kidney tumor cell lines, LAM 621-101 (human, Research Resource Identifier [RRID]: CBCL_S897) (28), 105K (mouse) (65), and TMKOC (mouse) (66), were provided by Drs Jane Yu and Elisabeth Henske. TMKOC was originally generated by Dr Vera Krymskaya (67). HEK293E cell line (RRID: CVCL_6974), MCF7 (RRID: CVCL_0031), BT549 (RRID: CVCL_1092), and UMB1949 (RRID: CVCL_C471) were obtained from American Type Culture Collection. LAM 621-101, UMB1949, MCF7, BT549, TMKOC, 105K, and HEK293E cells were grown in Dulbecco's modified Eagle's medium (GIBCO) with 10% fetal bovine serum (FBS) (Sigma–Aldrich) at 37 °C with 5% CO_2_. About 5 × 10^6^ cells counted by Multisizer 4e Coulter Counter (Beckman) were plated on a 60 mm plate and serum starved for 24 h unless otherwise indicated. Rapamycin (Calbiochem) dissolved in dimethyl sulfoxide (DMSO) was treated at the final concentration of 20 nM (LAM 621-101, UMB1949, MCF7, and BT549) or 100 nM (TMKOC and 105K). MAPK13-IN-1 (MAPK13 inhibitor; MedChemExpress) dissolved in DMSO was treated at the final concentration of 5 μM unless otherwise indicated.

### Transfection of DNA and siRNA

siRNAs (Sigma–Aldrich) dissolved in nuclease-free water were transfected into cells using Lipofectamine RNAiMAX reagent (Invitrogen) at the final concentration of 30 nM. siRNA list is provided in Table S1. For expression of the human siRNA-resistant mouse Mapk13 plasmid, pCDNA3-FLAG-Mapk13 (Addgene; catalog no.: 20785) (57) was transfected using FuGENE HD (Promega) 2 days before siRNA transfection.

### Cell proliferation assay

siRNA-transfected cells were seeded on a 60 mm plate. After 24 h, cells were treated with DMSO (control) or rapamycin without FBS. For cotreatment of MAPK13 inhibitor with rapamycin, MAPK13-IN-1 was pretreated 1 h before rapamycin unless otherwise indicated. Cell numbers were measured using Multisizer 4e Coulter Counter (Beckman) at 0 and 72 h after treatment. Cell proliferation (fold change) was calculated by dividing the cell numbers at 72 h by the cell numbers at 0 h.

### Cell migration assay

Wound-healing assay was applied to assess cell migration. siRNA-transfected cells were seeded on a 6-well plate. After 24 h, cells were treated with DMSO (control) or rapamycin without FBS. Once cells are confluent, a clear wound line was created using a sterile 200 μl pipette tip. Cell images containing the wound area were taken at 0 and 24 h using Eclipse Ts2-FL microscope and DS-Fi3 Camera (Nikon). Cell migration efficiency (%) was calculated by measuring the cell migration area (0–24 h) using the ImageJ software program (NIH).

### Crystal violet assay

Cells grown on 12-well plates were fixed with 4% methanol-free formaldehyde (Polysciences) and incubated with 0.1% crystal violet solution (Sigma–Aldrich) for 30 min. After rinsing five times with PBS, the plates were scanned for image analysis. For quantification, crystal violet dyes were eluted from the cells using methanol, and the absorbance of crystal violet solution was measured at 570 nm using Victor Nivo plate reader (PerkinElmer).

### Immunoblot

Cells were homogenized on ice using radioimmunoprecipitation assay lysis buffer (25 mM Tris–HCl [pH 7.4], 2 mM EDTA, 150 mM NaCl, 0.1% SDS, 2 mM DTT, 1% sodium deoxycholate, and 1% NP-40) supplemented with protease inhibitors (1 mM PMSF, 2 μg/ml pepstatin A, 10 μg/ml leupeptin, and 10 μg/ml aprotinin) and phosphatase inhibitors (10 mM NaF and 1 mM Na_3_VO_4_). Cell lysates were cleared by centrifugation at 13,000 rpm at 4 °C for 30 min. Detergent-compatible protein assay (Bio-Rad) was used to measure protein concentration. Proteins were boiled for 10 min with Laemmli sample buffer. SDS-PAGE gels were used to separate proteins (10–30 μg) and transferred to the nitrocellulose membrane (Amersham Biosciences). Membranes were then incubated with Odyssey blocking solution (Li-COR Biosciences), followed by incubation with primary and IRDye secondary antibodies (Li-COR Biosciences). Immunoblot signals were detected and quantified by Image Studio software with the Li-COR imaging system (Li-COR Biosciences). Immunoblot images are representative of at least two independent experiments. Primary antibodies against p-S6(S240/S244) (catalog no.: 2211), S6 (catalog no.: 2317), beta-actin (catalog no.: 3700), p38 (MAPK11/12/14) (catalog no.: 8690), METTL3 (catalog no.: 86132), METTL14 (catalog no.: 51104), Pan-Akt (catalog no.: 4691), p-Akt (S473) (catalog no.: 4060), eEF2 (catalog no.: 2332), p-eEF2 (T56) (catalog no.: 2331), YTHDF2 (catalog no.: 71283) and cMyc (cataolg no.: 5605) (Cell Signaling Technology); WTAP (catalog no.: Ab195380) (Abcam); and MAPK13 (catalog no.: AF1519) (R&D Systems) were used.

### Protein stability analysis

Cells were treated with 50 μg/ml cycloheximide (Sigma–Aldrich) to inhibit translation, and cell lysates were collected at 0, 1, 2, 4, 6, 12, and 24 h to analyze the remaining protein levels. Protein expression was analyzed by immunoblot assay as described previously.

### qPCR

PureLink RNA isolation kit (Life Technologies) was used to isolate total RNA from cells. After removing genomic DNA by DNase I (Sigma–Aldrich), RNA was reverse transcribed to complementary DNA using the iScript kit (Bio-Rad). The resulting complementary DNA was analyzed by qRT–PCR using SYBR Green Master Mix (Life Technologies) on QuantStudio6 Real-Time PCR system (Life Technologies). For the qPCR screen in Figure 1, 17 final candidate genes from our previous miCLIP-Seq performed in HEK293E cells with and without mTOR inhibitor, torin1, were used (24). mRNA levels were calculated by delta–delta CT method using housekeeping genes *ACTIN*, *PPIB*, and *TBP* (human), or *Actin*, *Tbp*, and *36B4* (mouse). The primer list is provided in Table S2.

### mRNA stability analysis

Cells were treated with 5 μg/ml actinomycin D (Sigma–Aldrich) to inhibit transcription and collected at 0, 4, and 8 h to analyze the remaining mRNA levels. Total RNA was extracted, and mRNA levels were analyzed by qPCR as described previously.

### Luciferase reporter assay

HEK293E cells were seeded on a 12-well plate. After 24 h, 500 ng of renilla (Switchgear Genomics S805935 MAPK13 3′UTR or MAPK13 m^6^A site mutant constructs) and 100 ng of cypridina (Switchgear Genomics SN0322S) luciferase constructs were cotransfected into cells using FuGENE HD (Promega). About 48 h after transfection, luciferase activity was measured using LightSwitch Renilla Luciferase Assay reagent (Switchgear Genomics) and Pierce Cypridina Luciferase Glow Assay kit (Pierce) on Victor Nivo plate reader (PerkinElmer) according to the manufacturer’s protocols. The activity of renilla luciferase was normalized by cypridina luciferase activity.

### Site-directed mutagenesis

The point mutation of m^6^A modification site (A1212 to T1212) of human MAPK13 3′UTR luciferase reporter (Switchgear Genomics S805935) was generated using a QuickChange site-directed mutagenesis kit according to the manufacturer’s protocol using Pfu Ultra polymerase (Agilent Technologies). Mutagenesis primers are MAPK13-3UTR-GGACC-mut-fw (5′-CACTGCCCAAGGTCCAGTATTTGTC-3′) and MAPK13-3UTR-GGACC-mut_rv (5′-GACAAATACTGGACCTTGGGCAGTG-3′).

### Analysis of sequence conservation

CDS and 3′UTR sequences of *MAPK13* were obtained from the National Center for Biotechnology Information database: human (NM_002754.5) and mouse (NM_011950.2). Sequence alignment was performed using Clustal Omega (EMBL-EBI).

### GEO dataset analysis

RNA-Seq results of rapamycin-treated UMB1949 cells were obtained from public dataset (GEO accession number: GSE193402). The raw fastq files were mapped to Ensembl human genome assembly GRCh38.107 using the STAR aligner (version 2.7.10b). Raw counts calculated from featureCounts (version 2.0.3) were used as inputs for Deseq2 (version 1.34) for the differential gene expression analysis.

### Statistical analysis

Statistical analyses were performed using GraphPad Prism software (GraphPad Software, Inc). All values are presented as mean ± SD. Statistical significance was determined using a two-tailed Student’s *t* test for comparison between two. Statistical significance is presented as ∗*p* < 0.05, ∗∗*p* < 0.01, ∗∗∗*p* < 0.001, or ns = not significant.

## Data availability

All data are included within the article and Supporting information. The materials and methods in this study are available from the corresponding author upon request.

## Supporting information

This article contains supporting information (24).

## Conflict of interest

The authors declare that they have no conflicts of interest with the contents of this article.

## Supporting information

Supporting Figure S1 **mRNA levels of miCLIP-seq candidate genes in rapamycin-treated UMB1949 cells (Related to**Fig. 1**).** In the public dataset GSE193402, RNAseq was performed in TSC2-deficient human renal angiomyolipoma cell line (UMB1949) treated with (control) or 50 nM rapamycin for 24 h. The mRNA level changes of our 17 miCLIP-seq candidate genes (24) are analyzed and presented on the graph. The Y axis denotes normalized fold change of RNAseq read counts. N = 3. ∗*p* < 0.05, ∗∗*p* < 0.01.

Supporting Figure S2 **Sequence alignment of human and mouse *MAPK13* (Related to**Fig. 3**).** (*Top*) Schematic of human *MAPK13* and mouse *Mapk13* mRNA. The percent identity (%) of the 5′UTR, CDS, and 3′UTR of human and mouse *MAPK13* were calculated using Clustal Omega. The percent identity of 57.7% in 3′UTR indicates that a 212 bp-long Mouse *Mapk13* 3′UTR can be aligned to the 5148 bp-long Human *MAPK13* 3′UTR with 57.7% similarity. (Bottom) Alignment of human *MAPK13* and mouse *Mapk13*‘s CDS and 3′UTR sequences around the m^6^A modification site (human A1212) identified from our miCLIP-seq analysis (24). The m^6^A consensus motif (GGACC) in human *MAPK13* is highlighted in *red*.

Supporting Figure S3 **Additional characterization of MAPK13 expression and MAPK13 inhibitor, MAPK13-IN-1 (Related to**Fig. 4**).***A*, QPCR analysis of LAM 621-101 cells transfected with siNTC or siMAPK13 in combination with DMSO or rapamycin treatment. *B*, crystal violet assay of LAM 621-101 cells treated with DMSO or rapamycin (20 nM) for 14 days in combination with a dose-dependent treatment of MAPK13-IN-1 (5 nM ∼ 20,000 nM). Relative cell growth (%) was calculated compared to the crystal violet absorbance of DMSO-treated cells. Note that 5 μM MAPK13-IN-1 shows the most synergistic effect with rapamycin in cell growth suppression (*red arrow*). N = 5. *C* and *D*, protein stability analysis of MAPK13 and cMYC. HEK293E cells were treated with cycloheximide (50 μg) for the indicated times and the remaining protein levels were measured by immunoblot. *D*, shows the quantification graph of immunoblot bands. N = 4. ∗∗*p* < 0.01, ∗∗∗*p* < 0.001. Error bars show standard deviation (SD). Numbers on the immunoblot indicate the positions of molecular weight markers.

Supporting Table S1 siRNA list.

Supporting Table S2 Primer list.
