# Supplementary figures and images for: MAPK13 stabilization via m6A mRNA modification limits anticancer efficacy of rapamycin
Source: J Biol Chem. 2023 Aug 19;299(9):105175. doi: 10.1016/j.jbc.2023.105175 (PMC10511813; doi:10.1016/j.jbc.2023.105175)

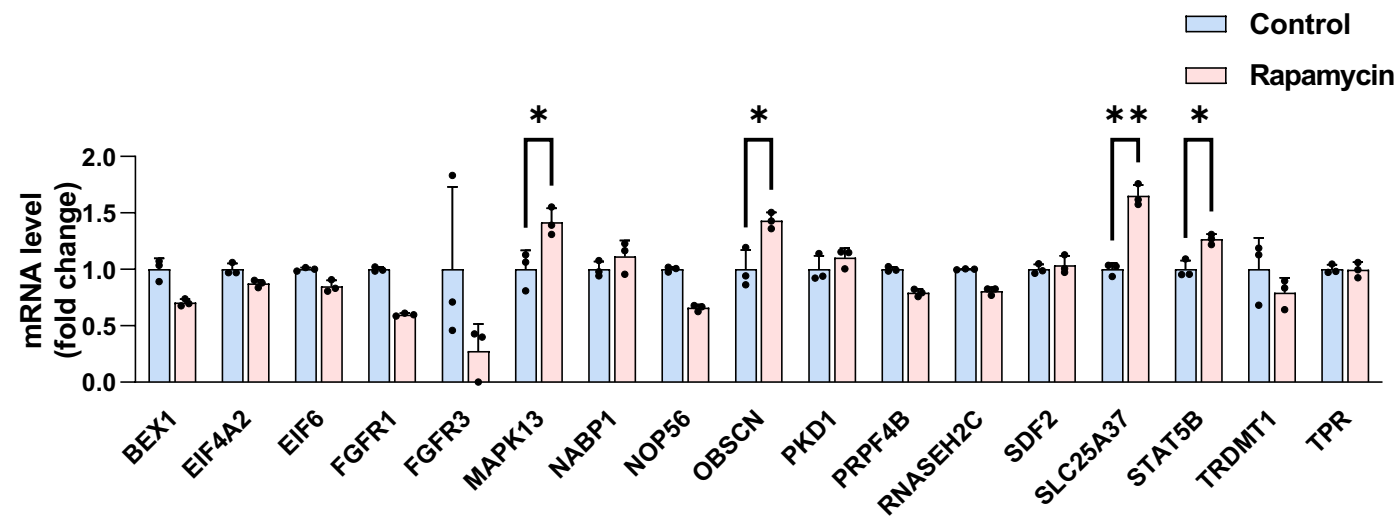

Supplemental Figure S1

Supplement: Supporting Figure S1 — mRNA levels of miCLIP-seq candidate genes in rapamycin-treated UMB1949 cells (Related toFig. 1). In the public dataset GSE193402, RNAseq was performed in TSC2-deficient human renal angiomyolipoma cell line (UMB1949) treated with (control) or 50 nM rapamycin for 24 h. The mRNA level changes of our 17 miCLIP-seq candidate genes (24) are analyzed and presented on the graph. The Y axis denotes normalized fold change of RNAseq read counts. N = 3. ∗p < 0.05, ∗∗p < 0.01. [file mmc1.pdf]

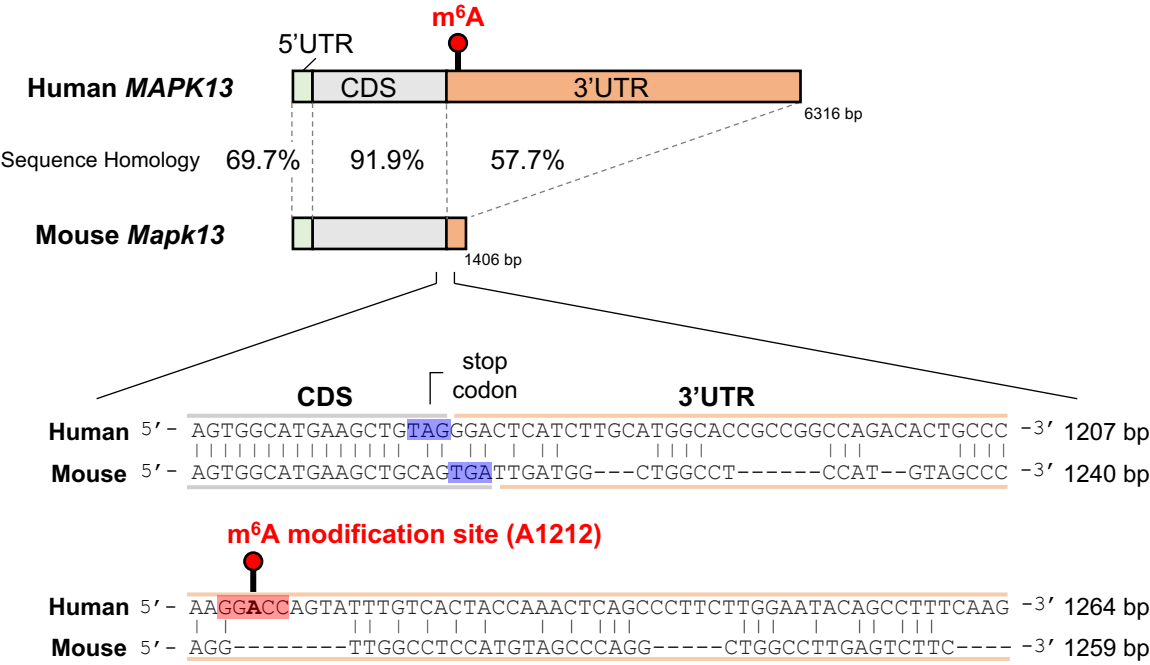

Supplemental Figure S2

Supplement: Supporting Figure S2 — Sequence alignment of human and mouse MAPK13 (Related toFig. 3). (Top) Schematic of human MAPK13 and mouse Mapk13 mRNA. The percent identity (%) of the 5′UTR, CDS, and 3′UTR of human and mouse MAPK13 were calculated using Clustal Omega. The percent identity of 57.7% in 3′UTR indicates that a 212 bp-long Mouse Mapk13 3′UTR can be aligned to the 5148 bp-long Human MAPK13 3′UTR with 57.7% similarity. (Bottom) Alignment of human MAPK13 and mouse Mapk13‘s CDS and 3′UTR sequences around the m6A modification site (human A1212) identified from our miCLIP-seq analysis (24). The m6A consensus motif (GGACC) in human MAPK13 is highlighted in red. [file mmc2.pdf]

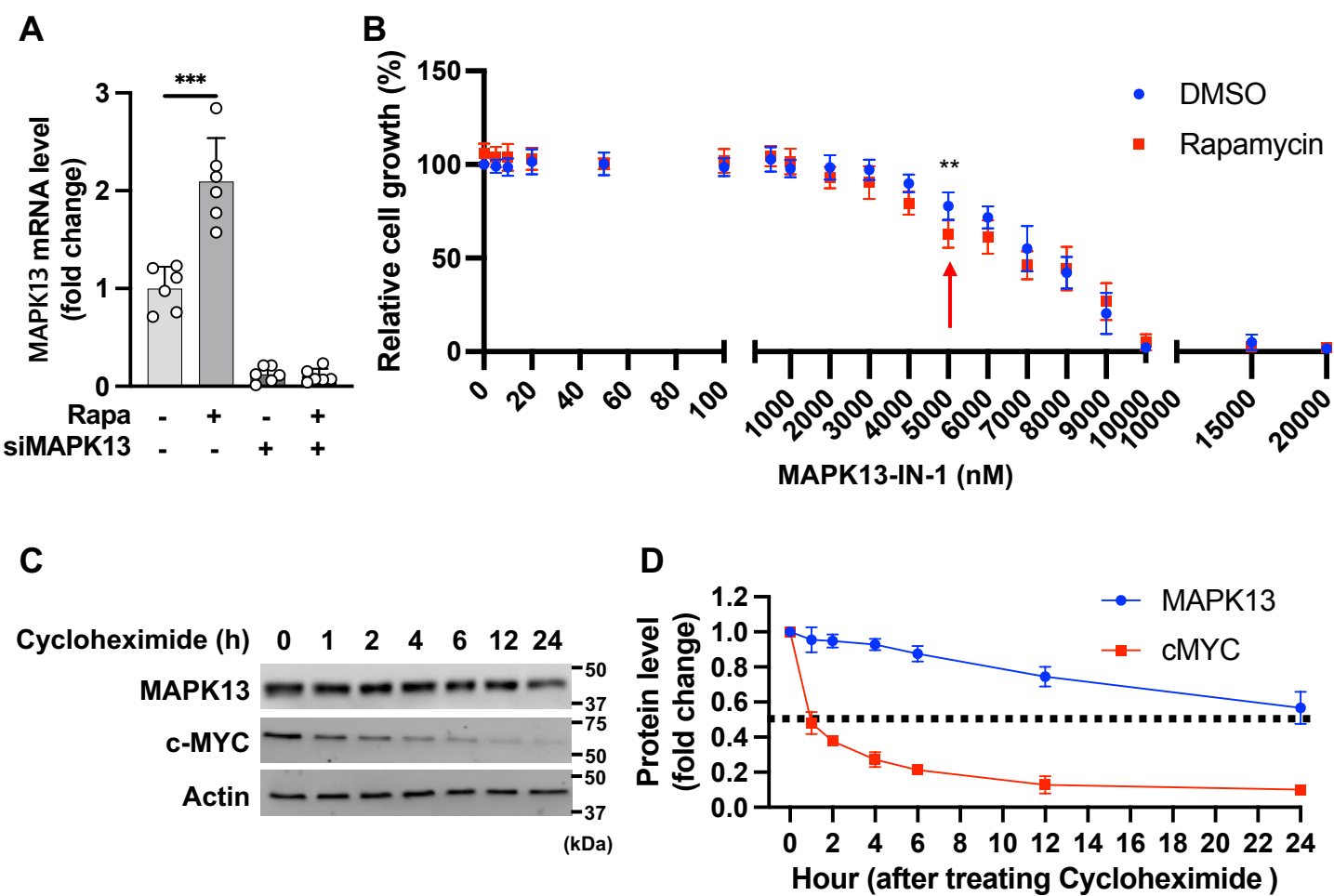

Supplement: Supporting Figure S3 — Additional characterization of MAPK13 expression and MAPK13 inhibitor, MAPK13-IN-1 (Related toFig. 4).A, QPCR analysis of LAM 621-101 cells transfected with siNTC or siMAPK13 in combination with DMSO or rapamycin treatment. B, crystal violet assay of LAM 621-101 cells treated with DMSO or rapamycin (20 nM) for 14 days in combination with a dose-dependent treatment of MAPK13-IN-1 (5 nM ∼ 20,000 nM). Relative cell growth (%) was calculated compared to the crystal violet absorbance of DMSO-treated cells. Note that 5 μM MAPK13-IN-1 shows the most synergistic effect with rapamycin in cell growth suppression (red arrow). N = 5. C and D, protein stability analysis of MAPK13 and cMYC. HEK293E cells were treated with cycloheximide (50 μg) for the indicated times and the remaining protein levels were measured by immunoblot. D, shows the quantification graph of immunoblot bands. N = 4. ∗∗p < 0.01, ∗∗∗p < 0.001. Error bars show standard deviation (SD). Numbers on the immunoblot indicate the positions of molecular weight markers. [file mmc3.pdf]
